# Noradrenergic neurons regulate the egress and trafficking of splenic monocytes and influence mortality during Gram-negative infection in mice

**DOI:** 10.1186/cc11758

**Published:** 2012-11-14

**Authors:** EJ Seeley, S Barry, M Matthay, PJ Wolters

**Affiliations:** 1 University of California San Francisco, CA, USA; 2 Stanford University, Palo Alto, CA, USA

## Background

Neurotransmitters derived from the autonomic nervous system can regulate inflammatory cytokine secretion. This has been extensively studied in the parasympathetic nervous system, where acetylcholine regulates the secretion of TNF from splenic macrophages during LPS-induced inflammation. However, the role of noradrenergic neurons during mouse models of infection has not been characterized. The goal of these experiments was to study the influence of noradrenergic neurons on the immune response during Gram-negative septic peritonitis in mice.

## Methods

Peripheral noradrenergic nerves were ablated using 6-hydroxydopamine (6-OHDA), a commonly employed method for studying noradrenergic neurons. Four days later, septic peritonitis was induced by i.p. injection of 150 CFU *Klebsiella pneumoniae*. Survival, serum and peritoneal bacterial loads, inflammatory cytokine production and leukocyte recruitment were studied at multiple time points after infection. To assess the importance of the NE containing splenic nerve, survival experiments on splenectomized mice with or without 6-OHDA treatment were performed.

## Results

**Figure 1:**
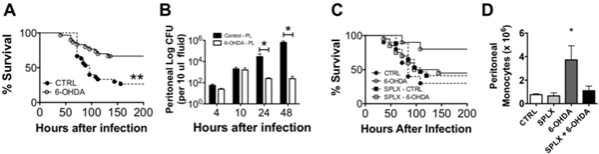
**Nonadrenergic nerve ablation with 6-OHDA improves survival during *K***. *pneumonia *intraperitoneal sepsis **(A)**. Nonadrenergic nerve ablation leads to improved bacterial killing **(B) **and enhanced monocyte recruitment 4 hours after infection **(D)**. The survival benefit of 6-OHDA treatment required the spleen because splenectomy (SPLX) prior to 6-OHDA treatment abolishes this survival benefit **(C)**. Peritoneal monocytes were quantified 4 hours after infection in 6-OHDA-treated mice with or without spleens (D). 6-OHDA treatment enhanced the recruitment of splenic monocytes to the peritoneum during infection (D). *P < 0.05.

Ablation of noradrenergic nerves improved survival following *K. pneumoniae *septic peritonitis (Figure 1A). Mice in which noradrenergic nerves had been ablated showed a more robust immune response 4 hours after infection with higher systemic IL-6, higher intraperitoneal MCP-1 and a fourfold increase in monocyte recruitment into the peritoneum. Bacterial loads were lower 24 and 48 hours after infection in mice in which noradrenergic nerves had been ablated (Figure 1B). Four hours after infection, 6-OHDA-treated mice recruited more inflammatory monocytes to the infected peritoneum (Figure 1D, CTRL vs. 6-OHDA). Splenectomy prior to noradrenergic nerve ablation abrogated the beneficial effects of 6-OHDA during infection (Figure 1C) and reduced the recruitment of monocytes to the infected peritoneum (Figure 1D, 6-OHDA vs. SPLX + 6-OHDA).

## Conclusion

These results suggest that splenic nerve-derived catecholamines regulate the egress of splenic monocytes during infection and that altering this pathway can alter survival during septic peritonitis in mice. These data highlight the emerging role of splenic monocytes during inflammation and infection [1] and add to the body of evidence that the immunosuppressive effects of catecholamines can impair innate immune responses during infection [2]. These experiments may have important implications for patients receiving vasopressors in the ICU.
